# Nucleic Acid Nanomaterial-Mediated Single-Cell Encapsulation and Its Application

**DOI:** 10.3390/bios15110712

**Published:** 2025-10-27

**Authors:** Yue Qiu, Mengyu Huang, Xiaotong Jiang, Peiru Chen, Zhenzhen Guo, Kaixiang Zhang

**Affiliations:** School of Pharmaceutical Sciences, Zhengzhou University, Zhengzhou 450001, China; qiuyue0518@gs.zzu.edu.cn (Y.Q.); myhuang@gs.zzu.edu.cn (M.H.); jiangxiaotong@gs.zzu.edu.cn (X.J.); chenpeiru@gs.zzu.edu.cn (P.C.)

**Keywords:** single-cell encapsulation, nucleic acid nanomaterial, cell protection, cell therapy, cell transplantation

## Abstract

Single-cell encapsulation, by constructing cell-scale microenvironments, enables precise protection, regulation, and functional enhancement of individual cells, holding significant importance in biomedical fields such as bioanalysis and cell therapy. Although various materials—including polymers, nanoparticles, hydrogels, polyphenols, and inorganic minerals—have been explored for single-cell encapsulation, limitations in controllability, biocompatibility, and multifunctional integration remain. In contrast, DNA nanomaterials offer unique advantages, including programmable architecture, high biocompatibility, precise spatial control, and modular functionality, making them highly suitable for the development of intelligent single-cell encapsulation systems. In this review, a systematic summary of recent advances in DNA nanomaterial-based single-cell encapsulation is presented. The fundamental encoding and assembly principles underlying the engineered encapsulation of cells at the membrane interface using DNA nanostructures are elucidated. Subsequently, the distinctive merits of DNA-based cell encapsulation and its applications in biomedical research are comprehensively summarized. Finally, the prevailing challenges and future directions in this burgeoning field are critically discussed, aiming to provide novel insights and perspectives for the advancement of advanced functional materials in both academic and clinical research pertaining to single-cell encapsulation.

## 1. Introduction

The encapsulation of individual living cells represents a groundbreaking frontier in bioengineering and materials science, enabling the construction of protective microenvironments with tailored physicochemical characteristics at the single-cell level [1,2,3,4]. Such a strategy has revolutionized the way cellular protection, manipulation, and functional enhancement are approached, with implications that permeate across regenerative medicine, precision diagnostics, cell therapy, and synthetic biology [5,6,7,8]. In recent years, this concept has evolved from passive shielding mechanisms to intelligent, stimuli-responsive systems capable of adapting to dynamic biological contexts [9,10,11].

Historically, materials employed for single-cell encapsulation—such as synthetic polymers [12,13,14,15], natural hydrogels [16,17,18,19,20,21], inorganic coatings, and metal–organic frameworks—have made important contributions by providing physical protection and enabling localized modulation of the cell microenvironment [22,23,24,25,26]. However, these conventional systems often suffer from key limitations that constrain their broader functionality and clinical translation. First, they typically lack dynamic responsiveness: most materials offer passive protection and cannot adapt their structure or permeability in response to biological cues [27]. Second, they exhibit limited functional modularity; integration of sensing, delivery, or actuation functions requires cumbersome chemical modifications and lacks precise spatial control. Third, the molecular resolution achievable with these materials is inherently coarse, making it difficult to organize or position functional moieties at the nanoscale. Additionally, many materials face issues of immunogenicity, degradation control, and cytotoxicity under physiological conditions [28,29]. As next-generation cell therapies, diagnostics, and synthetic biology tools increasingly demand intelligent, multifunctional encapsulation systems that operate at the molecular level, there is a critical need for materials that are both environmentally responsive and architecturally programmable. Ideally, such materials would offer tunable assembly, programmable disassembly, logic-gated responsiveness to complex stimuli [30], and the ability to seamlessly interface with cellular membranes. In response to these challenges, a new class of encapsulation materials has emerged at the intersection of nanotechnology and nucleic acid chemistry—DNA nanomaterials.

DNA nanomaterials, with their unparalleled programmability, structural definition, and biochemical compatibility, have recently emerged as transformative candidates in the field of single-cell encapsulation [31,32]. Leveraging the intrinsic properties of nucleic acids—base-pairing fidelity, hierarchical self-assembly, and environmental responsiveness—DNA nanostructures allow the construction of highly ordered [33], dynamically reconfigurable architectures that can interface intimately with living cells. These features unlock the potential for highly specific membrane anchoring, stimuli-triggered release, real-time signal transduction [34,35,36], and even logic-gated therapeutic intervention at the level of individual cells. Moreover, unlike conventional materials, DNA nanomaterials offer the capacity to integrate sensing, actuation, and computation within the same nanoscale framework [37,38,39,40]. This integrative capability makes them ideal for next-generation theranostic platforms—capable of sensing pathophysiological cues, delivering therapeutic payloads, and reporting cellular responses simultaneously. As such, DNA-based single-cell encapsulation systems not only serve as protective coatings but also as intelligent, multifunctional devices that dynamically interact with their cellular cargo and the surrounding biological environment.

**Figure 1:**
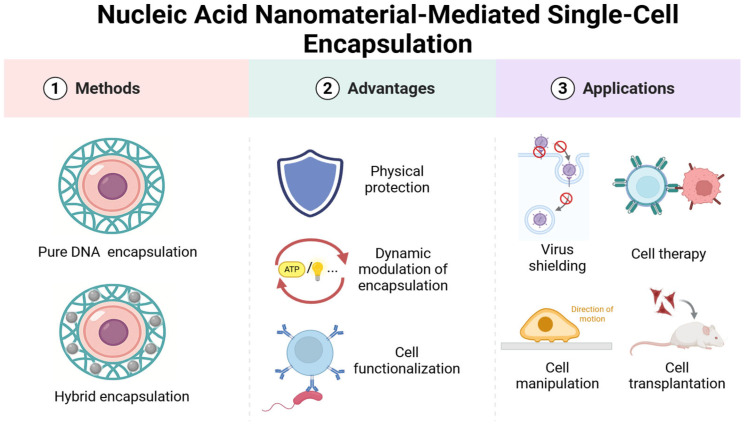
Illustration of nucleic acid nanomaterial-mediated single-cell encapsulation and its applications.

This review aims to systematically explore the emerging paradigm of single-cell encapsulation using DNA nanomaterials. The examination begins with the foundational principles underlying the design and assembly of DNA nanostructures at the cellular interface, including sequence-encoded programmability, multivalent interactions, and spatially organized surface engineering. The unique advantages of DNA-based systems over conventional encapsulation technologies are then highlighted, emphasizing their adaptability, molecular precision, and functional modularity. Subsequently, a broad range of biomedical applications—from precision drug delivery and immunomodulation to regenerative medicine and real-time bioanalysis—are explored, where DNA-encapsulated single cells are poised to make significant impacts. Finally, current challenges are discussed, and future directions are charted to accelerate the translation of this technology from conceptual frameworks to practical tools in clinical and research settings, see Figure 1.

## 2. Development of Cell Encapsulation Materials

Single-cell encapsulation materials can be broadly categorized into three main types based on their material properties: electrolyte polymers (such as poly-L-lysine (PLL)), polymeric materials (such as gels), and inorganic materials (such as silica). Among these, electrolyte polymers demonstrate unique application potential in single-cell encapsulation due to their tunable charge interactions and mechanical properties.

Polyelectrolyte polymers, materials like polylysine and chitosan, typically form encapsulating layers through electrostatic adsorption with anionic cell surface components, achieving cell protection [41,42,43]. For example, Sun et al. [44] prepared cross-linked gelatin nanocapsules by means of layer-by-layer (LbL) assembly of oppositely charged gelatin and combining it with transaminase-catalyzed cross-linking reactions, successfully constructing gelatin encapsulation layers on human cervical cancer cell lines (HeLa) and mouse insulinoma cell lines 6 (MIN6). HeLa and MIN6 cells exhibited high survival rates of 95.17 ± 11.04% and 86.93 ± 4.33%, respectively. The nanocapsules maintained stability for two days, significantly enhancing cellular resistance to cytotoxic enzymes (e.g., trypsin). Additionally, the Pires-Santos group studied alginate and poly-L-lysine microcapsules with selective permeable membranes to support the diffusion of essential nutrients and the release of factors, enabling both suspension cells and adherent cells (THP-1 cell line and UCMSC, respectively) to maintain stable survival over seven days of culture, with no significant differences in metabolic activity, The DNA content within the microcapsules also remained stable [45]. Although polyelectrolyte materials have successfully maintained the morphology and viability of umbilical cord mesenchymal stem cells for up to one week [46], and the alginate microcapsule coating significantly inhibits pericapsular fibrosis and inflammatory cell infiltration in microencapsulated islet transplantation, this may represent a promising technology for allogeneic islet transplantation and isograft islet transplantation with lower immune responses than xenogeneic islet transplantation [12,44,47]. However, their performance is highly dependent on environmental pH and ionic strength. For example, certain layers (such as gelatin/hyaluronic acid) disintegrate due to charge neutralization at extreme pH levels (e.g., 3.0 and 11.0) [43], and for self-assembled multilayer membranes containing poly(dimethyl diallyl ammonium chloride) and polyacrylic acid, the LbL membrane undergoes decomposition when ionic strength increases to a certain concentration [12,48]. Additionally, the positive charges of polycations (such as PLL), polyethyleneimine directly interact with the negative charges of cell membranes, leading to membrane rupture or pore formation at high concentrations or high molecular weights [7,41,42].

Additionally, gel materials, due to their three-dimensional network structure, exhibit high hydrophilicity, mechanical properties similar to the stiffness of the cellular extracellular matrix, and tunable force-biochemical characteristics, making them an important area of exploration [49,50,51,52,53]. Gel-mediated single-cell encapsulation primarily refers to the use of natural or synthetic polymeric materials (such as alginate, polyethylene glycol, agarose, etc.) to form micron-scale hydrogel networks, which then encapsulate individual cells using techniques such as microfluidic emulsification, photopolymerization, or electrospinning. This technology aims to mimic the functions of the extracellular matrix, achieving synergistic protection through physical isolation, nutrient exchange, and signal transduction. For example, Zhang et al. anchored microbial transaminase to the outer surface of the cell membrane to catalyze the cross-linking of gelatin, forming a single-cell coating that blocks the activity of apoptosis factors (including the binding of tumor necrosis factor α (TNFα) to the tumor necrosis factor receptor), thereby conferring stress resistance to mesenchymal stem cells (MSCs) and protecting them from TNFα-induced apoptosis [21]. Additionally, the microgel shell can isolate immune cell attacks and reduce shear force damage [16,54]; and by regulating gel stiffness and degradation rates, it can control cell proliferation and differentiation [18]. However, issues such as cell sedimentation, mixing uniformity, and insufficient dynamic response speed exist in large-scale production [50,54]. These defects have prompted researchers to explore new strategies, such as nucleic acid materials.

Although gel materials can provide a primary protective barrier through physical encapsulation, their mechanical stability and chemical functionality remain limited. In contrast, inorganic nanomaterials, with their high mechanical strength and assembly properties, offer a more robust physical barrier for single-cell encapsulation. This strategy involves constructing inorganic encapsulation layers (e.g., SiO_2_, MOFs) on the cell surface through biomineralization or coordination, forming a physical barrier to resist external stress. For example, Zhu et al. constructed an exoskeleton encapsulated in zeolite imidazolate framework-8 (ZIF-8) nanoparticles, which could freeze ZIF-8 through tannin-mediated particle-to-particle bonding in just 30 s of incubation [55]. Although the SupraCells technology utilizes ZIF-8 nanoparticle exoskeletons to enable mammalian cells to tolerate extreme osmotic pressure and UV radiation, the silanization process may induce cells into an irreversible dormant state, and maintaining cell activity remains a challenge in mammalian cell encapsulation. Long-term silanization may lead to irreversible inhibition of cell proliferation [56,57,58].

The above discussion of electrolyte polymers, gels, and inorganic materials reveals the current dilemma in single-cell encapsulation technology: material functionality and biocompatibility are often difficult to achieve simultaneously. For example, while electrolyte polymers offer advantages such as low cost and processability, their electrostatic adsorption mechanism is susceptible to environmental ion interference and may even induce cytotoxicity; hydrogels can mimic the extracellular matrix to provide three-dimensional support, but their static cross-linked networks limit dynamic response speed; and inorganic materials (such as silica) possess exceptional mechanical strength but may irreversibly inhibit cell proliferation.

Meanwhile, the immense potential and versatility of cell nano-encapsulation technology across various applications have driven its evolution from the first-generation passive shells, which were solely used as cell encapsulation layers, to the second-generation active shells. These second-generation active shells enable cells to exhibit biochemical reaction versatility while retaining the shell characteristics of the first-generation shells [27]. However, while this field does provide a useful platform for the development of cell therapy and single-cell biology research, it remains in its infancy, with shell-forming strategies for mammalian cells facing significant limitations in terms of cell compatibility, sustainability, and reliability [29].

In recent years, DNA nanotechnology has emerged as a promising and versatile tool for engineering cell membrane surfaces [59,60,61,62,63], offering a novel approach to overcoming the aforementioned limitations. Nucleic acids not only enable precise engineering modifications of single-cell interfaces through base-pairing molecular mechanisms but also inherently possess biocompatibility, significantly reducing material toxicity risks. Additionally, the sequence-function coupling properties of nucleic acid materials allow them to integrate multiple functions such as protection, response, and therapy into a single system—a capability that traditional materials struggle to match. The following sections will focus on nucleic acid-mediated single-cell encapsulation technology, exploring its classification, construction, advantages, and applications in detail.

## 3. Design and Construction of DNA-Mediated Cell Encapsulation

The sequence programmability and dynamic self-assembly properties of DNA materials have opened up innovative avenues for cell encapsulation technology. In recent years, DNA-based cell encapsulation systems have provided a new research paradigm for the field of biomedical engineering due to their unique molecular recognition capabilities and structural designability. These systems typically target cell membranes through cholesterol modification or nucleic acid aptamer binding, followed by the formation of protective DNA shells via base complementary pairing or hybridization chain reaction (HCR). Notably, this strategy can be further enhanced by integrating nanoparticles or functional molecules to achieve enhanced protection and multifunctional modification of cells. Given the prominent advantages of DNA-mediated cell encapsulation technology in precise regulation, biocompatibility, and functional expandability, it holds significant potential for development in synthetic biology and cell therapy. The following sections will provide a detailed overview of the classification, design principles, and construction methods of this strategy.

### 3.1. Nucleic Acid-Mediated Single-Cell Encapsulation Classification

In terms of composition, nucleic acid-mediated single-cell encapsulation can be divided into two main categories: pure DNA encapsulation shells and mixed DNA encapsulation shells. In mixed DNA encapsulation shells, DNA primarily serves as a scaffold during the formation of the encapsulation shell, capable of binding metal ions, electrolyte polymers, and nanoparticles to enhance protection and functionalization.

### 3.2. Pure DNA Material-Mediated Encapsulation Strategy

**Figure 2:**
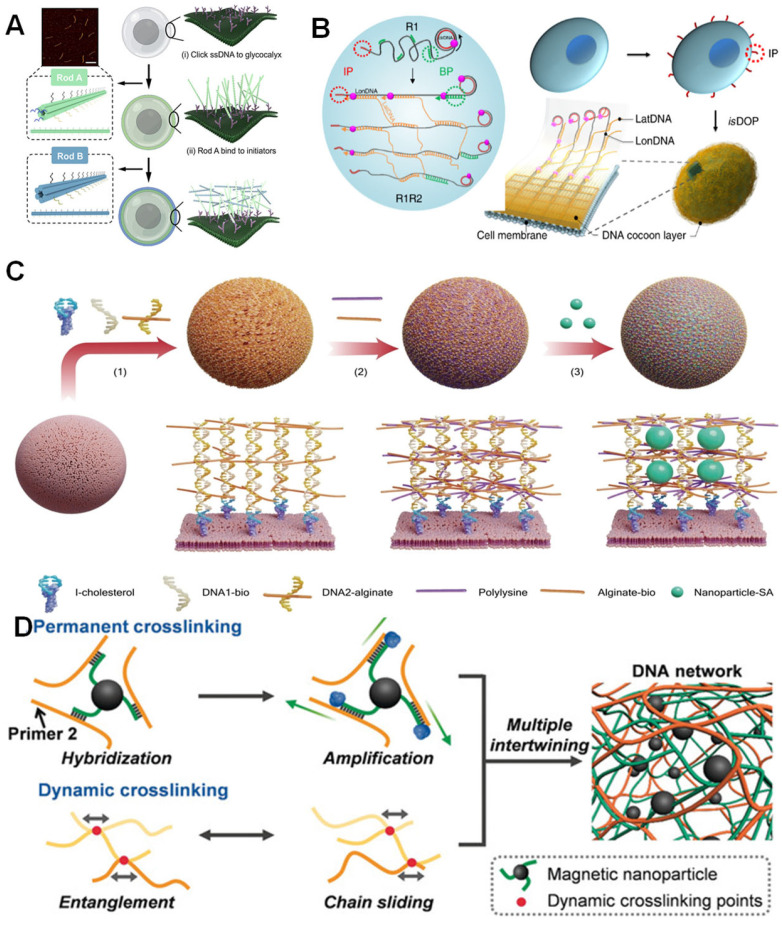
Assembly of DNA-programmed cell encapsulation. (**A**) Schematics of the nanoshell synthesis process and validation of stability [65]. Copyright 2023, American Chemical Society. (**B**) In situ DNA-oriented polymerization reaction for cell encapsulation [66]. Copyright 2019, Springer Nature. (**C**) Illustration of the carry nanoparticles (CN^2^) method [69]. Copyright 2023, Wiley-VCH. (**D**) Synthesis route of the magnetic DNA hydrogel [70]. Copyright 2020, Wiley-VCH.

DNA is prone to degradation in physiological environments [64], so in cell encapsulation, DNA is designed to form more complex three-dimensional nanostructures, such as polyhedra, origami, and nanogels. Wang’s research group designed double-layered cross-linked DNA nanorods (Rod A and Rod B), utilizing single-stranded DNA (ssDNA) on Rod A to target cell surface glycans and forming rigid nanocapsules through complementary hybridization with Rod B, while programming precise cellular assembly. This structure directly enhances membrane stiffness, reduces lipid fluidity, and modulates the biophysical properties of the cell membrane. The nanocapsules also act as armor to enhance the cell’s tolerance to mechanical stress (Figure 2A) [65]. Additionally, Gao et al. developed an in situ DNA-oriented polymerization reaction to attach the initial primer (IP) to the cell membrane. Then, rolling circle replication reactions, replication 1 and replication 2, guide the assembly of DNA cocoons on the cell surface. Specifically, IP initiates R1, which produces long, periodic DNA polymers (referred to as longitudinal DNA, LonDNA) when single-stranded circular DNA is introduced as a replication template. Subsequently, the branched primer initiates replication 2, generating a second single-stranded DNA polymer (referred to as latitudeDNA and LatDNA), leading to connection-based assembly across these initial polymers according to the replication template design. LonDNA and LatDNA automatically cross-assemble during replication, resulting in the in situ formation of DNA cocoons on the cell surface. Coating of bacterial, yeast, and mammalian cells has been achieved (Figure 2B) [66]. In contrast, another technique developed by the Fan group uses DNA framework nucleators (DFN) to guide the growth of DNA hydrogels under cell-friendly conditions. The DFN consists of a tetrahedral DNA framework structure with initiator DNA and cholesterol at its vertices. Compared to single-stranded DNA nucleating agents, tetrahedral DFN can anchor more stably and uniformly on the cell membrane, thereby effectively forming uniform, flexible cell encapsulation via HCR. This encapsulation maintains cell viability and protects cells from autophagy induced by mechanical stress [67]. Additionally, our group constructed programmable DNA scaffold networks using two functional modules: DNA trihedra (TP) are fixed to the hydrophobic interactions of the cell membrane lipid bilayer via cholesterol-labeled DNA chains; one-dimensional branched polymers (BP) are synthesized via HCR, with their length regulated by the ratio of initiators to monomers. By combining single-layer or multi-layer TP with BP (e.g., TP-BP, TP-mBP, and mTPs-BP), the structural tunability of DNA scaffold networks was achieved. The tunable network provides a simple and scalable strategy that addresses the current challenge of precisely manipulating cell-cell interactions in cell surface engineering using pure nucleic acid tools [68].

### 3.3. DNA-Based Composite Co-Encapsulation System with Other Substances

DNA nanostructures can combine with relevant biomolecules to further enhance their protective capabilities and even acquire new functions. Moreover, their combination with different substances endows them with distinct properties. Lee et al. encapsulated cells using a supramolecular DNA-alginate-polylysine composite and decorated them with nanoparticles (NPs) to construct a nano-biomimetic extracellular matrix. Initially, the DNA initiator was anchored to the cell membrane via cholesterol insertion. This was followed by the initiator triggered the assembly of DNA 1 and DNA 2 to form DNA nanostructures. Consequently, alginate and polylysine were crosslinked with the DNA nanostructures through electrostatic interactions, while NPs were immobilized within the three-dimensional space of the biomimetic nanocapsules via conjugation (Figure 2C). This system not only enabled the loaded nanoparticles to achieve photothermal gene regulation but also preserved the high metabolic activity of the cells, demonstrating the functional synergy between DNA, polymeric materials, and inorganic nanoparticles [69]. In terms of dynamic responsiveness, Tang et al. developed a magnetic DNA hydrogel robot by integrating the dynamic crosslinking network of DNA hydrogels with magnetic nanoparticles (MNPs) modified with short ssDNA (Figure 2D). Through the synergistic effects of chain entanglement and permanent magnetic crosslinking points, the robot achieved shape adaptability and magnetically driven navigation, making it suitable for cell delivery in confined spaces [70]. These composite systems expand the application boundaries of encapsulation technology by combining DNA as a primary scaffold with the physicochemical properties of other materials.

## 4. Advantages of DNA-Mediated Cell Encapsulation

Based on the base-pairing and dynamic self-assembly properties of DNA molecules, cell encapsulation technology utilizing DNA has constructed a highly controllable system for regulating the cellular microenvironment. Compared with traditional encapsulation strategies that rely on physical embedding or chemical cross-linking, DNA-mediated encapsulation systems can achieve a multi-dimensional upgrade from static protection to dynamic functional empowerment. Their advantages can be summarized at three levels: basic protection—forming a dense barrier through the precise arrangement of DNA nanostructures to effectively isolate external mechanical stress or biologically toxic molecules; responsiveness—utilizing DNA strand displacement reactions or environment-sensitive conformational changes to perceive and respond in real time to temperature, environmental small molecules, or specific biological signals; functionality—leveraging the editability of DNA sequences to directionally integrate targeted recognition, thereby endowing cell encapsulation with precise spatiotemporally controllable functions. The following will provide a detailed discussion centered around these three core advantages.

### 4.1. Basic Physical Protection

**Figure 3:**
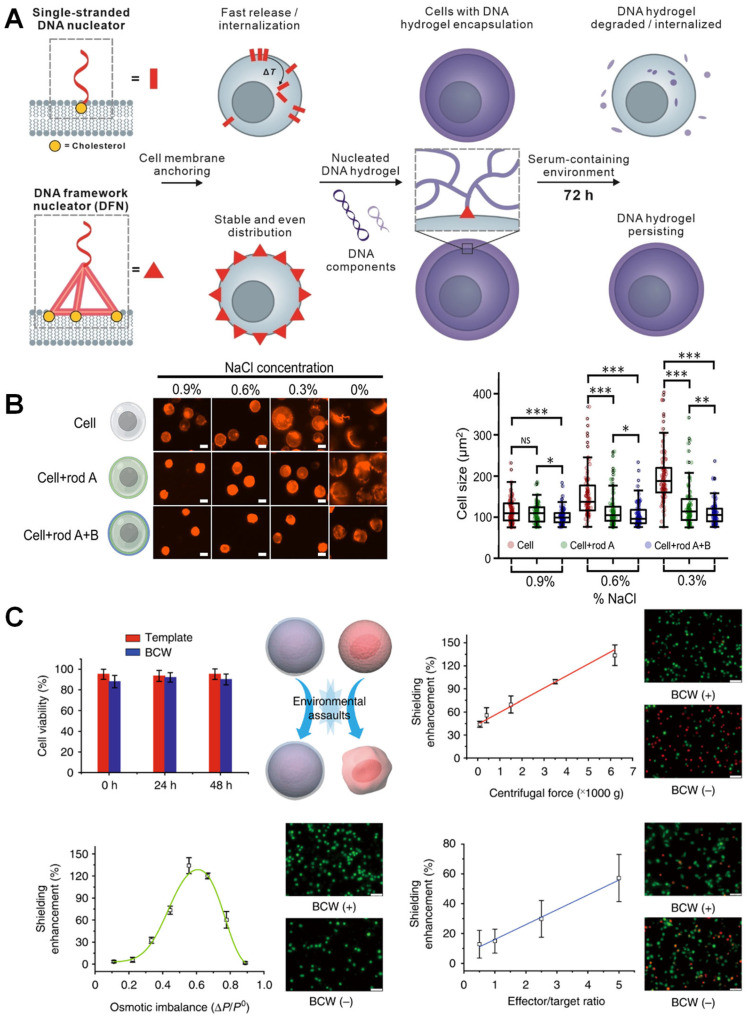
Fundamental protection in DNA-based cell encapsulation. (**A**) Schematic illustration for the construction of DNA coatings mediated by a DNA framework nucleator [67]. Copyright 2024, Wiley-VCH. (**B**) Protective effects of DNA nanoshell armor against challenging environments [65]. * *p* ≤ 0.05, ** *p* ≤ 0.01, *** *p* ≤ 0.001, and ns (not significant). Copyright 2023, American Chemical Society. (**C**) Evaluation of shielding enhancement [71]. Copyright 2019, Springer Nature.

Nucleic acid materials are prone to degradation, and when used as cell encapsulation materials, enhancing the stability of the encapsulation layer is of paramount importance. In 2024, DFN technology studied by Fan’s group pioneered a cholesterol multivalent modification strategy. Its rigid three-dimensional structure achieves homogeneous anchoring to the cell membrane through geometric matching effects, triggering HCR to form a degradable hydrogel encapsulation layer. This design extended the serum half-life to 72 h, and through fluorescence labeling of the LC3 protein, it was observed that the autophagy level of encapsulated cells was comparable to that of the untreated group, while autophagy in naked cells significantly increased under mechanical stress (Figure 3A) [67]. Additionally, the stability of the encapsulation layer was extended to the in vivo environment—Shi et al. employed a DNA template to crosslink an alginate–polylysine complex. The fluorescence intensity of BCW remained nearly unchanged over 48 h with the treatment of 1% FBS, ultimately leading to a significantly improved survival rate of encapsulated mesenchymal stem cells in a mouse model over a seven-day period [71].

Research on cell viability under extreme conditions has evolved into a core dimension for evaluating fundamental cellular protection. Wang’s research team investigated the protection of cells in harsh and mechanically challenging environments using a double-layered DNA nanorod-crosslinked nanoshell. As osmotic pressure decreased, the size of natural cells rapidly increased, and their viability declined. In contrast, nanoshell-coated cells maintained their size and exhibited approximately 20% higher cell viability in hypotonic solutions, showing a statistically significant difference compared to pretreated natural cells (Figure 3B). This showcases its ability to protect cells in challenging environments and its potential benefits for bioengineering applications, such as cell printing and multicellular assembly [65]. Additionally, Shi et al. developed a biocompatible wrapping (BCW) technology based on a polylysine-alginate framework template, where polylysine primarily reacts with alginate on the DNA template without affecting the cell plasma membrane. In physical stress tests, BCW-wrapped cells exhibited significant advantages: in terms of centrifugal force tolerance, they maintained a survival rate of approximately 85% after high-speed centrifugation at 6000× *g*, whereas naked cells lost over 30% viability during cyclic washing and centrifugation at 110× *g*. Regarding osmotic pressure adaptability, BCW demonstrated a shielding effect that enhanced the osmotic imbalance relationship, presenting a bell-shaped curve. When the osmotic pressure imbalance changed from 0.1 to 0.6, BCW could protect the coated cells. As the osmotic pressure imbalance further increased, BCW continued to shield the cells, although this shielding effect began to diminish. Additionally, the protective efficacy of BCW against biological attacks was validated using an natural killer NK-92 MI cell killing model, where BCW almost linearly improved the survival rate of target cells from NK-92 MI attacks (Figure 3C) [71].

### 4.2. Dynamic Response of Single-Cell Encapsulation

The core of single-cell dynamic response encapsulation technology lies in constructing an intelligent system with environmental adaptability. By leveraging precisely and remotely controlled stimulus-responsive systems, it can identify and react to changes in the pathophysiological microenvironment [72], thereby overcoming the limitations of traditional static encapsulation. This field has given rise to various response types, including chemical response, nuclease responsiveness, and physical stimulus response, which will be elaborated upon in the following sections.

**Figure 4:**
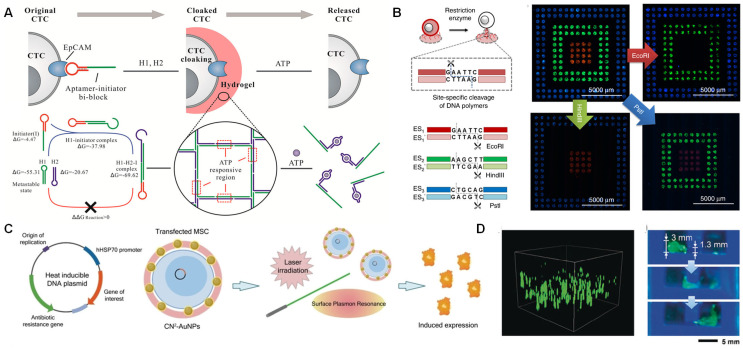
Dynamic response in single-cell encapsulation. (**A**) DNA gelation-based cloaking and decloaking of CTCs [73]. Copyright 2017, American Chemical Society. (**B**) Postediting of the DNA polymer cocoons for precise handling of cells [66]. Copyright 2019, Springer Nature. (**C**) Examination of cell regulation [69]. Copyright 2023, Wiley-VCH. (**D**) DNA robot as a vehicle for cell delivery [70]. Copyright 2020, Wiley-VCH.

Chemical response systems regulate cell encapsulation behaviors through stimulus-responsive DNA sequences. External stimuli such as metal ions, pH levels, and ATP concentrations can act as triggers for nucleic acid structures. Examples include metal ion-bridged duplexes, single strands (such as DNAzymes and i-motifs, etc.), triplex nucleic acids, G-quadruplexes, or programmed duplex hybrids of oligomers. These stimulus-responsive components provide functional scaffolds for the rapidly evolving field of DNA nanotechnology [59]. Fan’s research group employed an aptamer-triggered clamped hybridization chain reaction method for the encapsulation of circulating tumor cells (CTCs). Following ATP response, the morphological change of the aptamer from an unfolded state to a tertiary state compelled the collapse of the DNA hydrogel, thereby enabling the release of tumor cells (Figure 4A) [73].

In addition, enzymes are valuable molecular tools in DNA bioengineering, capable of “gluing,” “sawing,” or “trimming.” DNA ligase joins two DNA strands through covalent bonds, while endonucleases recognize and cleave specific DNA sites. Nuclease-responsive DNA editing systems enable precise cellular manipulation. Gao et al. successfully constructed an enzyme-responsive cellular encoding system by precisely integrating the recognition sequences (ES1-3) for EcoRI-HF, HindIII-HF, and PstI-HF restriction endonucleases into the circular template of DNA cocoons. Each ES1-3-encoded system demonstrated high release specificity for cells, with rates of 90.5%, 93.5%, and 98.0%, respectively, indicating the high specificity of targeted cell release (Figure 4B). Notably, this system achieved reversible control over the cleavage process—enzyme treatment dissociated only the outer cleavable structures while preserving the cell-anchoring function mediated by the initiating primer. Furthermore, the released cells exhibited relatively high viability during subsequent proliferation culture. Therefore, by encoding and precisely editing specific DNA cocoons at high resolution, cells can be manipulated with precision [66].

Finally, physical response systems achieve precise manipulation through exogenous stimuli such as light and magnetism. A representative work in the field of light-controlled systems is the nucleic acid biomimetic nanocapsules. Lee et al. innovatively coupled the photothermal effect of gold nanoparticles with the heat shock protein promoter to enhance gene expression, and the natural retention rate of the nanoparticles reached 83.1% within 4 h (Figure 4C) [69]. Additionally, Tang et al. synthesized ultralong ssDNA using rolling circle amplification technology catalyzed by phi29 DNA polymerase. By hybridizing short ssDNA modified on the surface of MNPs with the ultralong ssDNA, permanent cross-linking was formed, endowing the DNA network with magnetic properties conferred by the MNPs. This property enabled movement under an applied magnetic field, with a cell viability rate of 92.15% (Figure 4D) [70].

In addition, there is a discernible trend in the developmental trajectory, evolving from single-stimulus response to multi-cascade regulation. Leveraging the unique structural transition properties of DNA aptamers and the toehold-mediated strand displacement reaction, You et al. designed a DNA-based device termed the “nano-claw.” This claw is capable of performing logic-based autonomous analysis of multiple cancer cell surface markers and, in response, generating diagnostic signals and enabling targeted photodynamic therapy [74]. Looking ahead, DNA-mediated cell encapsulation can achieve more precise regulation by drawing on the design principles of such multi-responsive DNA nanoelements.

### 4.3. Functionalization of Single-Cell Encapsulation

Nucleic acid-mediated single-cell encapsulation technology is evolving from a simple physical barrier to a multifunctional integration approach. Taking tumor immunotherapy as an example, this technology necessitates the simultaneous fulfillment of multiple functions, such as cell protection and targeted recognition, to effectively enhance the specific recognition between immune cells and cancer cells [75], ultimately improving therapeutic outcomes. Notably, aptamers, a class of DNA or RNA molecules capable of highly specific recognition of diverse targets, encompassing small organic molecules, inorganic substances, and even proteins of varying scales [76,77,78,79], hold significant application value in this technology.

**Figure 5:**
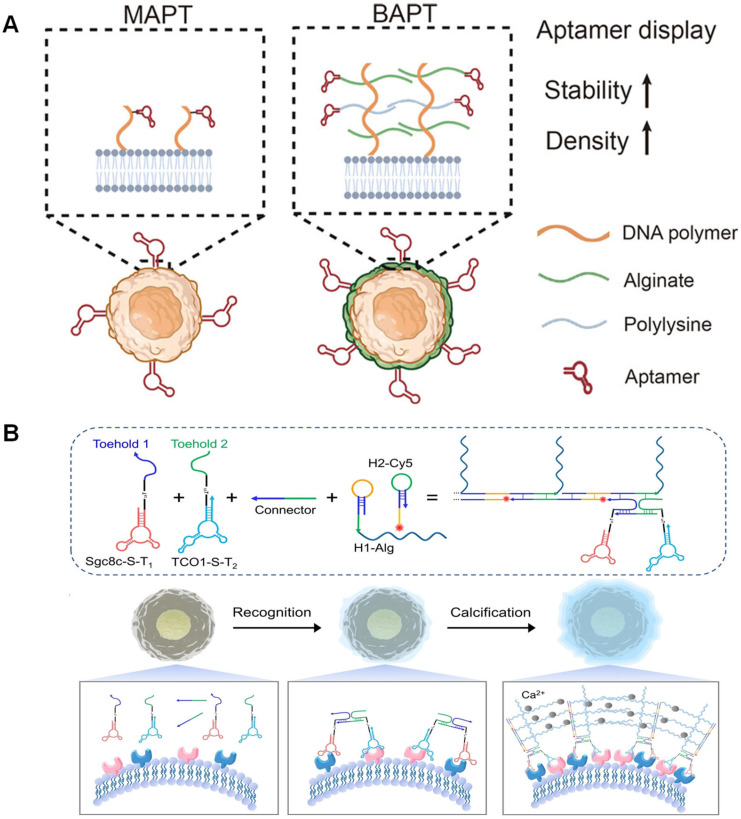
Functionalization of single-cell encapsulation. (**A**) Synthesis of an ultrathin alginate-polylysine coating for aptamer display on living cell surfaces, using NK cells as a model [80]. Copyright 2023, American Chemical Society. (**B**) The auto-assembled resilient biomimetic calcified ornaments (ARMOR) strategy was created by dual-aptamer-based HCR and Ca^2+^ assisted calcification for selective cell protection [81]. Copyright 2023, Wiley-VCH.

For instance, the biomimetic bacterial capsule coating technology developed by Wang et al. in 2023 demonstrated the potential of this strategy in immune cell engineering. By inserting DNA initiators into the NK cell membrane and triggering HCR, they successfully constructed a DNA–alginate–polylysine composite coating modified with nucleic acid aptamers. This technology enhanced the killing efficiency of NK cells against target cells by approximately twofold without compromising cell viability or the secretion of cytokines such as IL-12. Moreover, the coating significantly extended the half-life of nucleic acid aptamers on the cell membrane surface from less than 1.5 h to over 20 h, providing a stable platform foundation for the subsequent integration of functional modules (Figure 5A) [80]. Yu’s team proposed a self-assembling elastic biomimetic calcified shell strategy based on a dual-aptamer-triggered HCR and sodium alginate-induced calcification, which specifically assembled an elastic biomimetic armor on the surface of target cells for live cell encapsulation. The cell recognition strategy based on dual nucleic acid aptamers improved the selectivity of target cell recognition and enhanced the sensitivity of cell recognition through the in situ signal amplification reaction of HCR (Figure 5B) [81].

Addressing the challenges in constructing functionalized modules in existing single-cell encapsulation technologies, future innovations could integrate nucleic acid dynamic regulation with the construction of artificial organelles. Compared to the recent use of thiol-maleimide bond-mediated nano-encapsulation technology to achieve enzyme functionalization of lipid bilayers on the cell surface [82]. In the future, through a nucleic acid aptamer-mediated targeted anchoring system, dynamic regulation is achieved via DNA hybridization chains or RNA-protein interactions, while precise regulation is enabled by nucleic acid logic gates. By coupling pH- or ATP-sensitive DNA hairpin structures to the liposome surface, the encapsulation system can real-time sense changes in the extracellular microenvironment. This nucleic acid–liposome hybrid system not only retains the stabilizing effect of lipid bilayer assembly on enzyme activity but also achieves precise spatial arrangement of multiple composite enzymes through cascading nucleic acid switches. This paves the way for constructing a multi-functional integrated cellular protective system.

## 5. Biomedical Applications of DNA-Mediated Single-Cell Encapsulation

The multiple advantages of the nucleic acid-mediated single-cell encapsulation technology mentioned above provide an unprecedented single-cell manipulation platform for biomedical research. By using nucleic acid molecules as “smart carriers” that bind to the surface of single cells and respond to microenvironments, this technology can be applied in a variety of scenarios, ranging from antiviral and cell therapy enhancement to the precise encapsulation and capture of tumor vaccines, as well as improving cell transplant survival rates and other key areas. The following sections will discuss these application scenarios in detail.

### 5.1. Antiviral Physical Barrier Technology

**Figure 6:**
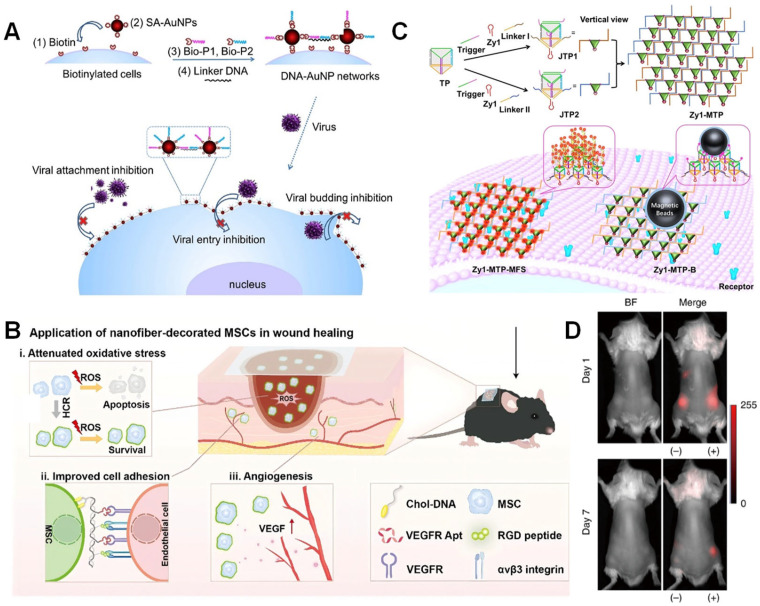
Diverse biomedical applications of single-cell encapsulation. (**A**) Schematic representation of the formation of DNA-AuNP networks on cell membranes and their inhibition behavior on viral attachment, entry, and budding [84]. Copyright 2016, Elsevier Ltd. (**B**) DNA nanofiber-engineered mesenchymal stem cells for wound healing [85]. Copyright 2024, American Chemical Society. (**C**) Illustration of DNA structures for the detection of tumor cells [86]. Copyright 2024, Wiley-VCH. (**D**) Evaluation of shielding enhancement [71]. Copyright 2019, Springer Nature.

Single-cell encapsulation technology has demonstrated unique advantages in the field of antiviral research, with its core strategy being the construction of physical barriers to block viral interaction with host cells. Two groundbreaking studies in 2016 revealed the dynamic protective mechanism of DNA-AuNP networks: Li’s team utilized a biotin-streptavidin directed anchoring system to cross-link gold nanoparticles into a network structure on the cell membrane surface, significantly interfering with key viral processes such as adsorption and entry through steric hindrance effects, while maintaining cell viability. Notably, compared to the traditional “one-to-one” inhibition mode targeting viral proteins, this physical barrier exhibits broad-spectrum antiviral properties (Figure 6A) [83,84]. However, the clinical translation of this technology still faces dual challenges: the enzymatic degradation of the DNA scaffold by DNase may weaken barrier stability, while the prolonged presence of the nano-network may impair cell membrane fluidity. To address these limitations, future efforts should focus on developing more stable DNA modifications and dynamically responsive barrier designs, thereby charting a path for the development of next-generation antiviral technologies.

### 5.2. Cell Therapy

In the field of cell therapy, single-cell protection technology enhances the survival rate of therapeutic cells through surface engineering and achieves functional enhancement of therapeutic cells. Taking MSC therapy as an example, in 2024, Wang et al. utilized DNA nanofiber self-assembly technology to anchor cholesterol-labeled Y-shaped DNA molecules to the MSC surface via membrane insertion within 10 min, triggering a cascade assembly reaction between Arg-Gly-Asp (RGD) peptide-modified hairpin structures 1 and VEGFR aptamer-modified hairpin structures 2 over 2.5 h, forming an integrated protective layer on the cell surface that combines RGD peptides and VEGFR aptamers (Figure 6B). These engineered F-MSCs enhance cellular function through the following mechanisms: (1) RGD peptide-mediated integrin-specific binding significantly enhances MSC adhesion to endothelial cells; (2) VEGFR aptamers promote neovascularization in injured areas by targeting and regulating vascular endothelial growth factor signaling pathways; and (3) the inherent antioxidant properties of DNA nanostructures confer ROS scavenging capacity, alleviating local oxidative stress microenvironments. Experimental results demonstrate that engineered F-MSCs exhibit significantly enhanced functionality: survival rates under oxidative stress conditions are significantly higher than those of N-MSCs (control group), endothelial cell-targeted adhesion efficiency is significantly improved, and wound healing in mice is accelerated. This non-genetic modification strategy effectively avoids the safety risks associated with traditional gene editing. These findings highlight the potential of DNA as a powerful strategy to overcome obstacles, enabling multiple functional components (such as small molecules, peptides, proteins, aptamers, etc.) to be precisely “labeled” on biological membranes as needed, thereby achieving personalized customization of cells and extracellular vesicles [85].

### 5.3. Precise Encapsulation and Capture of Tumor Cells

In recent years, non-invasive liquid biopsy technologies based on CTCs have developed rapidly. Luo et al. reported a novel strategy for tumor cell detection and separation based on spatially controllable DNA frameworks. The team self-assembled Janus DNA triangular prism nanostructures (3Zy1-JTP-3) and their multivalent hand-in-hand connected structures, enabling capture of target cells in whole blood with over 90% efficiency. A key advantage lies in the spatial characteristics of the triangular prism: the fluorescence intensity induced by 3Zy1-JTP-3 is nearly four times that of the monovalent structure. Furthermore, linking DNA triangular prisms forms a hand-in-hand multivalent DNA triangular prism structure, where fluorescence intensity and affinity increase to 9-fold and 10-fold, respectively, compared to 3Zy1-JTP-3. This work provides a novel solution for efficiently capturing rare cells in complex clinical biological samples (Figure 6C) [86]. Additionally, in the field of tumor vaccine development, single-cell encapsulation technology has achieved a technological leap from passive encapsulation to active recognition. In 2022, Yang et al. reported an aptamer-guided MOF encapsulation strategy: they functionalized precursor molecules with nucleolin aptamer AS1411 to specifically recognize tumor markers, forming a ZIF-8 protective shell through in situ mineralization under mild conditions. This mechanically enhanced shell significantly enhances antigen presentation efficiency by inducing immunogenic cell death, achieving improved tumor prevention rates in melanoma models, and reinforcing systemic immune responses through prolonged lymph node retention time (>48 h) and activation of the TNF/TLR pathway. Notably, while this study confirms the remarkable potential of the structure in tumor immunoprevention, critical gaps remain regarding its application boundaries. Further, the research only assessed the vaccine’s preventive efficacy against primary tumors; its therapeutic impact on established tumors and its ability to inhibit metastatic lesions still require validation in future investigations. Moreover, although the ZIF-8 shell demonstrated favorable biocompatibility under experimental conditions, expanding its clinical applicability will necessitate the exploration of MOF materials with enhanced biodegradability and more controllable immunomodulatory properties. This represents an essential direction for advancing this platform [87].

### 5.4. Cell Transplantation

Addressing the bottleneck issue of improving the survival rate of allogeneic cell transplantation, immune isolation technology based on biocompatible wrapping has demonstrated significant advantages. Shi et al. found that when human bone marrow MSCs expressing red fluorescent protein (RFP) were transplanted into a subcutaneous model of BALB/c mice, the allogeneic human bone marrow MSCs were rapidly destroyed by the host immune response. Seven days post-transplantation, almost no RFP signal was detectable in mice transplanted with bare MSCs. In contrast, RFP intensity remained at 30% in mice transplanted with MSCs covered by BCW. This significant difference strongly demonstrates that BCW can protect human MSCs from biological attacks in the in vivo environment (Figure 6D). This provides a new strategy for functional cell transplantation in regenerative medicine, avoiding attacks by the immune system and significantly improving the survival rate of transplanted cells [71]. Although this study successfully demonstrated the effectiveness of BCW in resisting physical and biological attacks, its clinical translation prospects still face critical challenges. On one hand, the long-term stability of BCW within the complex enzymatic environment of the body—particularly for applications requiring “permanent” shielding, such as islet transplantation—remains to be thoroughly investigated. On the other hand, the potential impact of BCW on the core functions of encapsulated cells, including their metabolic activity, intracellular signaling, and intercellular communication, necessitates systematic and comprehensive evaluation.

### 5.5. Applications of Biosensing

#### 5.5.1. Stable Membrane Interface Encapsulation for In Situ Monitoring

**Figure 7:**
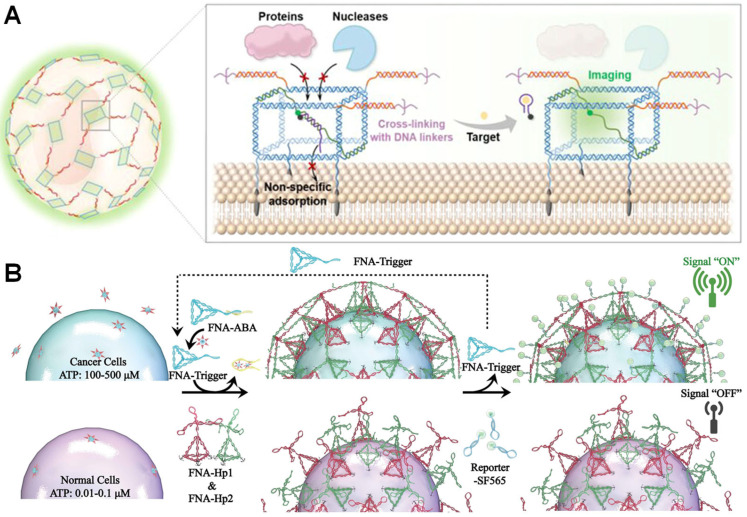
Single-cell encapsulation for biosensing applications. (**A**) Schematic Diagram of Imaging of Extracellular Targets in Biological Media Using PCCNTs [88]. Copyright 2025 American Chemical Society. (**B**) Cell surface-constrained FNATs for recognizing the extracellular microenvironment [89]. Copyright 2024, Wiley-VCH.

Achieving in situ dynamic monitoring of the extracellular microenvironment is crucial for deepening our understanding of cellular function and heterogeneity, while also imposing higher demands on probe stability within complex physiological settings. Traditional membrane-anchored probes are susceptible to interference in complex biological media, making reliable in situ detection challenging. To address this issue, Wang et al. developed a membrane interface sensing platform termed PCCNTs. This strategy employs programmable DNA cubes to efficiently encapsulate functional nucleic acid probes (e.g., ATP aptamers and G-quadruplex K^+^ probes), anchoring them to cell membranes via cholesterol modifications to construct stable sensing structures on single-cell membranes. This nucleic acid-mediated encapsulation not only effectively shields against nucleases in serum and plasma but also significantly enhances probe retention stability via in situ cross-linking. The platform demonstrates high-fidelity imaging of ATP and K^+^ released from individual cells in serum-containing media and plasma, showcasing potential for resolving cellular functions in complex physiological environments. However, challenges remain, including complex DNA structural assembly and the need for in vivo validation (Figure 7A) [88].

#### 5.5.2. Signal-Triggered Smart Encapsulation and Sensing Regulation

In nucleic acid-mediated single-cell encapsulation and sensing, achieving in situ manipulation responsive to extracellular signals is crucial for enhancing technological intelligence and functional integration. Inspired by the NETosis process, Gong et al. developed framework nucleic acid traps (FNATs) that enable in situ self-assembly and encapsulation triggered by specific molecular signals on target cell membranes. The core of this technology lies in utilizing framework nucleic acids (FNAs) pre-anchored to the cell membrane as a reactive foundation. Upon the presence of target molecules (such as ATP, highly expressed in the tumor microenvironment), the CHA reaction of the surface-anchored FNAs is triggered, dynamically forming a reticular DNA structure on the surface of individual cells. This achieves physical encapsulation and spatial confinement of the target cells. FNATs not only function as sensors, enabling visual identification of tumor-like microenvironments through fluorescence-on modes, but also effectively suppress the migratory capacity of encapsulated cells. Furthermore, this encapsulation structure can be further functionalized—for example, by loading photosensitizers to induce apoptosis—demonstrating the immense potential of nucleic acid-mediated encapsulation technology for integrating sensing and regulatory functions (Figure 7B) [89].

Nucleic acid-mediated single-cell encapsulation redefines cellular functional roles by precisely anchoring sensing interfaces to cell membranes. This transforms cells from passive “sensing elements” in traditional sensors into active, addressable, and even programmable “intelligent sensing units.” The core advantage of this technological paradigm lies in confining specific sensing events to the confined space on the surface of each encapsulated single cell. This spatial confinement effect effectively prevents diffusion and dilution of signal molecules in bulk solution, significantly enhancing signal-to-noise ratio and detection sensitivity through “localized enrichment.” Furthermore, the nucleic acid materials relied upon in this technology exhibit excellent biocompatibility and precise controllability. They provide a critical solution for achieving long-term, stable in situ dynamic monitoring at the living cell level, overcoming the stability limitations of traditional probes in complex physiological environments. In summary, nucleic acid-mediated single-cell encapsulation technology, through its unique spatial control, signal amplification, and biocompatibility, is emerging as a core driver propelling next-generation single-cell analysis sensing technology toward practical, high-throughput, and intelligent applications.

In summary, as a revolutionary technology that breaks through the natural functional limitations of cells, single-cell encapsulation technology demonstrates multi-dimensional application value in key fields such as antiviral therapy, regenerative medicine, and tumor immunology through bio-inspired engineering approaches. Its core innovation lies in establishing a precise interaction interface between the “cell surface and microenvironment,” enabling multi-level interventions ranging from molecular blockade to systemic regulation. Encouragingly, with the integration of dynamic DNA nanotechnology and smart responsive materials, single-cell encapsulation based on DNA materials is expected to open up more new fields.

## 6. Challenges and Future

Although significant progress has been made in the stability, dynamic response, and functionalization of nucleic acid-mediated single-cell encapsulation technology, it still faces multiple technical bottlenecks. First, there is a prominent contradiction between biocompatibility and stability: while DNA nanostructures offer programmable advantages, natural nucleases can easily cause degradation [90]. Although existing methods have managed to enhance nucleic acid encapsulation stability by constructing complex three-dimensional nanostructures or integrating microcapsule technology, where oppositely charged polyelectrolytes (such as chitosan/sodium alginate) are used for layer-by-layer self-assembly on the surface of DNA structures to form a semi-permeable membrane that effectively shields nucleases, there is a lack of a universally applicable and simple encapsulation method.

In addition, although DNA is generally considered biocompatible, overly rapid degradation of the carrier may lead to nucleic acid fragments triggering immune responses [91,92]. Moreover, a large amount of DNA on the cell surface may stimulate immune cells through the Toll-like receptor pathway in a manner similar to neutrophil extracellular traps [93,94,95,96,97]. This can be addressed by chemically modifying the nucleic acid backbone [98], such as using xenonucleic acids by replacing ribose with cyclohexane (e.g., locked nucleic acids) or morpholino groups to avoid recognition by the immune system as pathogen-associated molecular patterns. Furthermore, introducing phosphorothioate or methylation modifications can enhance stability through steric hindrance and enzyme resistance [99,100,101,102]. Second, the universality of functional modules is limited: aptamer targeting depends on specific membrane markers, resulting in insufficient coverage of heterogeneous cell populations, and the efficiency of matching precursor molecules with cell surface functional groups limits universal protection across multiple cell types. Therefore, it is necessary to discover more aptamers with high affinity and specificity for cell membrane proteins. Furthermore, the dynamic regulatory capability needs to be enhanced. While there have been numerous studies on nucleic acid-responsive systems, encapsulation systems still predominantly rely on static protective layers (such as DNA origami nanocapsules), lacking research on responsive dissociation in response to in vivo microenvironmental stimuli [103]. For better translation into practical applications, DNA-mediated encapsulation layers should be carefully tailored to adapt to the final functional environment, taking into account cell types and operational methods to improve batch stability and reduce the risk of cell damage. Additionally, machine learning should be integrated to assist in DNA sequence design and predict folding stability.

Challenges in Controllability and Reproducibility: Despite well-established design principles, DNA folding or assembly processes are prone to mismatches, hybridizations, or partial structural defects, which can compromise performance consistency. Therefore, substantial sequence optimization is required, which is relatively time-consuming and labor-intensive. In the future, AI-aided optimization could be employed to enhance efficiency. Furthermore, cell encapsulation based on DNA nanomaterials is still in its infancy. Achieving reversible and controllable encapsulation of individual cells using DNA nanomaterials, along with the subsequent intelligent manipulation of cellular behaviors (such as proliferation, migration, and directional movement) and the sensing of extracellular microenvironmental biomarkers, remains a significant challenge. Moreover, related clinical studies remain scarce, underscoring the necessity for continued and substantial efforts in these areas in the future.

## 7. Conclusions

In summary, the exceptional biocompatibility and programmability of nucleic acid molecules have opened up unprecedented technical opportunities in the field of single-cell encapsulation. Although nucleic acid-mediated single-cell encapsulation technology has achieved precise encapsulation at the single-cell level and demonstrated multiple advantages, such as physical barrier construction, dynamic response to microenvironments, and integration of functional modules, maintaining long-term stability, achieving multifunctional synergistic regulation, and scaling up production remain the core challenges currently faced. In the future, through the multidisciplinary integration of materials engineering, synthetic biology, and microfluidic technology, this technology is expected to undergo a paradigm shift from a “laboratory tool” to a “clinical-grade solution,” providing transformative technical support for fields such as cell therapy and single-cell analysis.

## Data Availability

No primary research results, software, or code have been included and no new data were generated or analyzed as part of this review.

## Abbreviations

The following abbreviations are used in this manuscript:

MIN6	Mouse insulinoma cell lines 6
PLL	Poly-L-lysine
TNFα	Tumor necrosis factor α
MSCs	Mesenchymal stem cells
ZIF-8	Zeolite imidazolate framework-8
HCR	Hybridization chain reaction
BCW	Biocompatible wrapping
IP	Initial primer
DFN	DNA framework nucleators
TP	DNA trihedra
BP	Branched polymers
NPs	Nanoparticles
MNPs	Magnetic nanoparticles
ssDNA	Single-stranded DNA
CTCs	Circulating tumor cells
NK	Natural killer
RFP	Red fluorescent protein
H	Hairpin structures
LonDNA	Longitudinal DNA
LatDNA	Latitude DNA
LbL	Layer-by-layer
ROS	Reactive oxygen species
RGD	Arg-Gly-Asp
CN^2^	Carry nanoparticles
ES	Recognition sequences
